# Assessing Clinical Coding Compliance in a Mental Health Inpatient Unit: An Audit and Intervention Study

**DOI:** 10.1192/bjo.2024.371

**Published:** 2024-08-01

**Authors:** Sirous Golchinheydari

**Affiliations:** West London NHS Trust, London, United Kingdom

## Abstract

**Aims:**

Clinical coding (CC) is the translation of medical terminology into a coded format that is recognised both nationally and internationally. NHS trusts must record the clinical care given to inpatients and the resources used for inpatients while they are in hospital care. CC ensures accurate patient records, communication and data exchange between providers and can aid in epidemiological research, healthcare planning and quality as well as cost control. An audit was carried out in a mental health inpatient unit to assess whether CC was completed as per the local and national CC guidelines, followed by an intervention to improve compliance.

**Methods:**

2 inpatient wards were identified, 1 male and 1 female, and 10 patients from each ward were selected at random on the 15^th^ of December 2023. Their notes were assessed to determine whether: the CC has been updated during their current admission, CC has been updated if new diagnosis, CC had been completed on last discharge, physical health conditions were included in the CC and the number of physical health diagnosis changes and their documentation. Intervention was carried out and a re-audit completed on the 31^st^ of January 2024.

**Results:**

Out of 20 patients: 5 (25%) had a completed CC during their admission and 4 had a diagnosis change but only 1 (25%) CC was updated. 9 had a physical health diagnosis but only 3 (33%) were included on CC. 16 (89%) had a completed CC on last discharge and 2 were admitted for the first time.

Doctors on the wards were informed about CC, how to access the form on the system and the importance of updating CC. This was communicated in teaching sessions and doctor communication groups.

The re-audit showed some improvement. Out of 20 patients: 10 (50%) had a completed CC during their admission, 4 had a diagnosis change and 3 (75%) CC were updated. 7 had a physical health condition and only 2 (29%) were included on CC. 12 (75%) had a completed CC on last discharge and 4 were admitted for the first time.

**Conclusion:**

The audit showed a lack of awareness of CC and its importance. The intervention helped to improve compliance of CC in current inpatients. Further intervention and improvement is required for physical health CC and can be attempted with posters in the doctor's rooms and regular reminding during group sessions.

